# Methionine cycle-dependent regulation of T cells in cancer immunity

**DOI:** 10.3389/fonc.2022.969563

**Published:** 2022-08-10

**Authors:** Tian Zhao, Julian J. Lum

**Affiliations:** ^1^ Trev and Joyce Deeley Research Centre, BC Cancer, Victoria, BC, Canada; ^2^ Department of Biochemistry and Microbiology, University of Victoria, Victoria, BC, Canada

**Keywords:** T cells, cancer, metabolism, the methionine cycle, cancer immunotherapy, immunemetabolism

## Abstract

The methionine cycle comprises a series of reactions that catabolizes and regenerates methionine. This process is crucial to many cellular functions, including polyamine synthesis, DNA synthesis, redox balance, and DNA and histone methylation. In response to antigens, T cells activate the methionine cycle to support proliferation and differentiation, indicating the importance of the methionine cycle to T cell immunity. In cancer, T cells serve as important effectors of adaptive immunity by directly killing cancerous cells. However, the tumor microenvironment can induce a state of T cell exhaustion by regulating the methionine metabolism of T cells, posing a barrier to both endogenous T cell responses and T cell immunotherapy. Here we review the role of methionine cycle metabolites in regulating the activation and effector function of T cells and explore the mechanism by which tumor cells exploit the methionine pathway as a means of immune evasion. Finally, we discuss new perspectives on reprogramming the methionine cycle of T cells to enhance anti-tumor immunotherapy.

## Introduction

### The methionine cycle and methionine-derived metabolites are crucial to cellular functions

The anti-tumor function of T cells is closely related to the metabolism of amino acids and other nutrients in T cells (1, 2). For example, elevated arginine metabolism leads to enhanced anti-tumor function of T cells whereas Kynurenine generated in tryptophan metabolism induces T cell exhaustion in the tumor microenvironment (TME) (3). Importantly, the uptake and utilization of methionine are crucial for the effector activity of T cells (4, 5), a key in adaptive immunity against cancer. The activation and function of T cells in the context of cancer immunity have been extensively discussed elsewhere (6). In this review, we focus on the roles of methionine metabolism specifically in T cells, and how this knowledge can be used to manipulate methionine to enhance T cell anti-tumor immunity.

**Figure 1:**
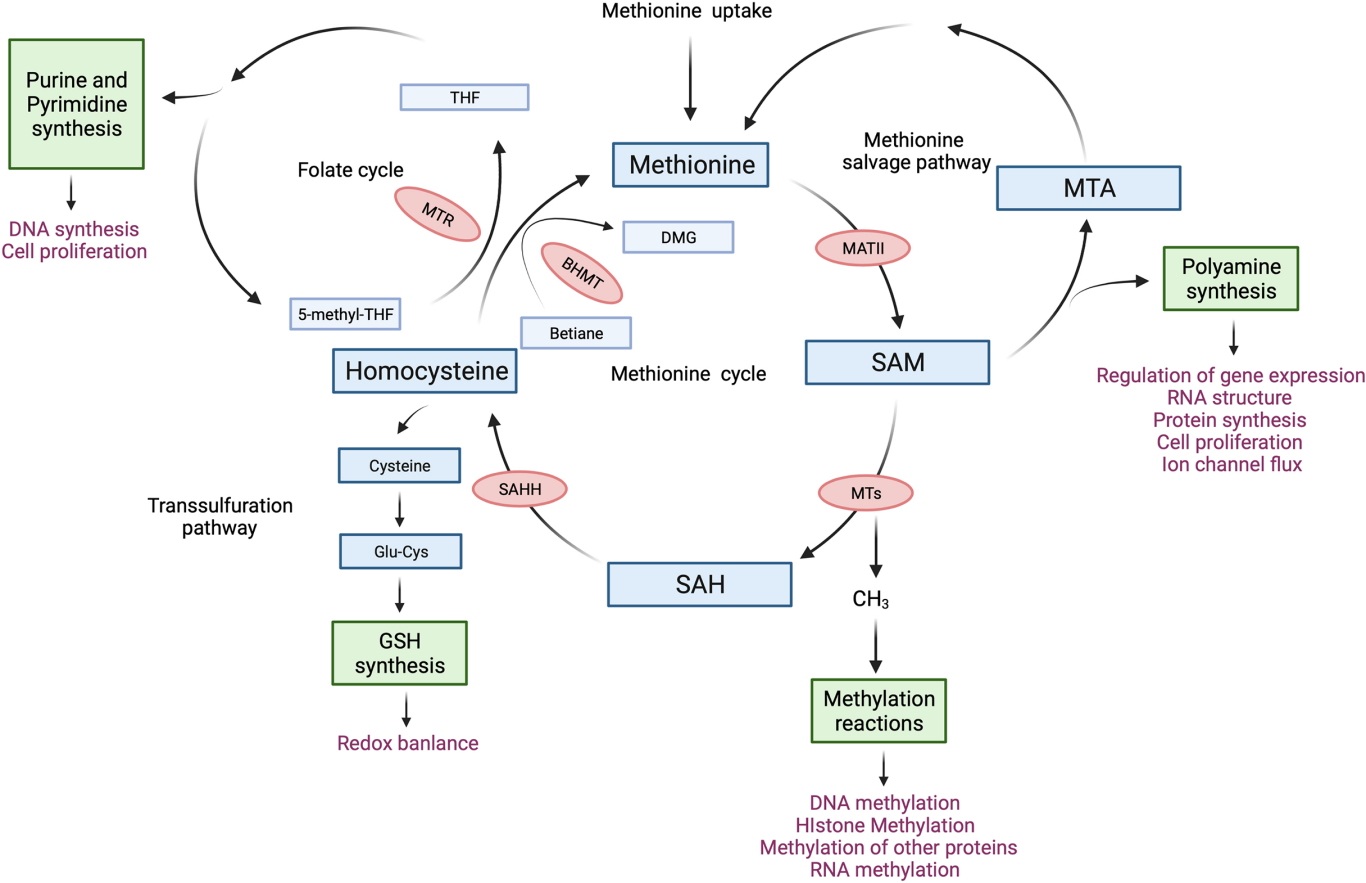
The methionine cycle in T cells. After entering T cells, methionine flows into the methionine cycle. The first step of the methionine cycle is the conversion of methionine into S-adenosylmethionine (SAM) by methionine adenosyltransferase II (MATII). SAM is converted into S-adenosylhomocysteine (SAH) after donating a methyl group for methylation reactions. This step is mediated by Methyltransferases (MTs). SAH is then hydrolyzed by S-adenosyl-L-homocysteine hydrolase (SAHH) to generate homocysteine. Finally, homocysteine receives a methyl group from the folate cycle or betaine to become methionine, these reactions are mediated by 5-methyltetrahydrofolate: homocysteine methyltransferase (MTR) and betaine-homocysteine methyltransferase (BHMT) respectively. The methionine cycle is interconnected with three important metabolic pathways by providing substrates. These pathways include the folate cycle, the transsulfuration pathway, the methionine salvage pathway, and polyamine synthesis, all of which support important cellular functions. DMG, dimethylglycine; 5-methyl-THF, 5-methyltetrahydrofolate; THF, tetrahydrofolate; Glu-Cys, γ-glutamylcysteine; GSH, glutathione; MTA, 5′-Methylthioadenosine.

Methionine is a sulfur-containing essential amino acid and is historically known as the first amino acid to be recruited to the ribosome to initiate protein synthesis in eukaryotes. Methionine depletion in culture media drastically suppressed protein synthesis in immortalized cell lines by impairing the recognition of translation start sites (7). More recent work on the metabolism of methionine has revealed its essentiality in other cellular functions. For instance, methionine produces metabolites that are key to polyamine synthesis, DNA synthesis, redox balance, and methylation reactions (8). The core of methionine metabolism is the methionine cycle which comprises a series of reactions that catabolize and regenerate methionine (**Figure 1**). The methionine cycle generates S-adenosylmethionine (SAM), which is a universal methyl group donor. SAM is required for methylation reactions including histone methylation and DNA methylation, in which it donates a methyl group and becomes S-adenosylhomocysteine (SAH). Importantly, changes in SAM and SAH levels regulate the kinetics of histone methylation marks (9, 10). Specifically, SAH is a potent inhibitor of DNA Methyltransferases (DNMTs) and Histone Methyltransferase (HMTs) whereby the cellular concentration of SAH determines the activity of methyltransferases (10). Moreover, regulating SAM and SAH levels by methionine restriction alters the level of histone methylation in CD4+ T cells (5). Thus, the abundance of SAM and SAH can shape the epigenetic landscape in T cells.

Another important product of the methionine cycle is homocysteine. Homocysteine connects the methionine cycle with the transsulfuration pathway. Homocysteine can either enter the transsulfuration pathway to produce glutathione (GSH) and control redox balance or be converted to methionine (**Figure 1**). The transsulfuration pathway contributes to proper T cell activation and proliferation through the upregulation of cystine and cysteine import (11–13). In line with this, knocking out glutamate-cysteine ligase (GCL), which catalyzes the rate-limiting step of the transsulfuration pathway, blocks the transsulfuration pathway, thus inhibiting the proliferation and altering the development of T cells (14, 15). Moreover, overexpression of cystathionine-γ-lyase (CTH), another enzyme in the transsulfuration pathway, in CD8+ T cells enhances their anti-tumor function. However, this did not affect GSH levels or cytokine expression in CD8+ T cells (16). Homocysteine also connects the methionine cycle with the folate cycle. The conversion of homocysteine to methionine not only completes the methionine cycle, but also contributes substrates to the folate cycle (**Figure 1**). The folate cycle is crucial for purine and pyrimidine synthesis and is required for the proliferation of CD8+ T cells (17) as well as the survival of regulatory T cells (18, 19).

### Methionine cycle machinery

As with most metabolites, methionine is closely regulated by an active transporter system. Several transporters are capable of transporting methionine into cells, including SLC1A5, SLC7A5, SLC7A6, SLC38A2, and SLC43A2 (8). SLC7A5 is expressed in both mouse CD4+ and CD8+ T cells (4, 38). Its expression in mouse CD4+ T cells is induced by T cell activation through CD3/CD28 engagement, which leads to increased methionine uptake (4). After entering T cells, methionine flows into the methionine cycle (**Figure 1**). The first step of the methionine cycle is the conversion of methionine into SAM by methionine adenosyltransferases (MATs). There are three MAT genes in mammalian cells, *MAT1A, MAT2A*, and *MAT2B*. *MAT1A* is expressed mainly in liver cells and encodes the catalytic subunit α1 (MAT1a), which forms MATI (homo-tetramer) and MATIII (homo-dimer) (20). *MAT2A* and *MAT2B* are expressed in all tissues, encoding the catalytic subunit α2 (MAT2a) and the regulatory subunit β (MAT2b), respectively (21). Two units of MAT2a and one unit of MAT2b form MATII, which has a lower Km than MATI and MATIII and is inhibited by MAT2b (20, 22). Importantly, MATs are found to be present in the nucleus (23, 24). Moreover, MAT2a can be recruited to nuclei to generate SAM locally and interact with HMTs (23), suggesting a possible role of MAT2a in regulating histone methylation by providing SAM locally to HMTs. MATII is important for T cell activation as mitogen-induced T cell activation induces the expression of *MAT2A* while downregulating the expression of *MAT2B* (25). By increasing the synthesis of SAM from methionine, T cells can support epigenetic remodeling that ultimately drives their differentiation (26, 27). SAM is converted into S-adenosylhomocysteine (SAH) after donating a methyl group for the methylation of a variety of cellular substrates, including DNA, RNA, phospholipids, histone proteins, and other proteins (28). SAH is then hydrolyzed by S-adenosyl-L-homocysteine hydrolase (SAHH) to generate homocysteine. As mentioned, homocysteine can either enter the transsulfuration pathway or receive a methyl group from the folate cycle or betaine to become methionine, the latter is mediated by 5-methyltetrahydrofolate: homocysteine methyltransferase (MTR) and betaine-homocysteine methyltransferase (BHMT) respectively (**Figure 1**). Methionine is therefore interconnected to other pathways and the multiple levels by which the level of methionine and methionine-related metabolites are controlled provide unique avenues that when modulated can have profound consequences on T cell function. Indeed, recent studies on the methionine cycle of T cells have revealed the important roles of the methionine cycle in regulating T cell immunity (**Table 1**), we discuss the findings of these studies in detail in the following sections.

## The methionine cycle-dependent regulation of T cells activation and effector function

### The methionine cycle in T cells

During activation, T cells undergo large-scale metabolic reprogramming to produce energy and metabolites for proliferation and exerting effector functions (40). The importance of the methionine cycle to T cells is underscored by its requirement for remodeling the histone methylation landscape during T cell differentiation (5). Histones can be monomethylated (me), dimethylated (me2), or trimethylated (me3). Such modifications can lead to repressed gene expression (e.g. H3K27me3, H3K9me3, and H3K9me2) or activated gene expression (e.g. H3K4me3 and H3K79me3) (41). During the development of T cells, H3K4me3 was upregulated in lineage-specific gene loci, e.g. Interferon-gamma (*IFNG*) locus in Th1 cells, whereas H3K27me3 and H3K9me2 were induced in the silenced lineage-promiscuous gene loci, e.g. interleukin-4 (*IL4*) and interleukin-17 (*IL17*) loci in Th1 cells, this facilitates the establishment of lineage-specific expression profile (42–44).

### Sustained methionine uptake is required for the activation of T cells

T cells undergo rapid proliferation and differentiation upon recognition of antigens. Exogenous methionine is required during this process to support protein synthesis and generate SAM for methylation reactions required for T cell activation. For example, arginine methylation of cytokine receptor γ-chain regulates the lineage commitment of T cells (45), and the modification of DNA methylation and histone methylation landscape is required for T cell function and differentiation (27, 41). For example, differentiation into CD4+ T cells is associated with an increase in 5-hydroxymethylcytosine (5hmC, promoting DNA demethylation) and a decrease in 5-methylcytosine (5mC) at lineage-specific gene loci. On the other hand, Th2 cells exhibited increased 5hmC and reduced 5mC at *GATA Binding Protein 3* (*GATA3*) and *IL4* loci whereas Th1 cells had increased 5hmC at *IFNG* and *T-Box Transcription Factor 21* (*TBX21*) loci (46, 47). Similarly, a gain of active histone methylation mark H3K4me3 in lineage-specific loci and an increase in repressive histone mark H3K27me3 and H3K9me2 in silenced lineage-promiscuous gene loci were observed during CD4+ T cell development (42–44). As a result, T cells have to sustain methionine influx during activation. In line with this, Sinclair et al. (4) found that mouse CD4+ T cells drive flux through the methionine cycle in response to antigen by upregulating the expression of methionine transporters (SLC7A5 and SLC1A5), thus permitting RNA methylation and the remodeling of H3K4me3 and H3K27me3 to promote T cell proliferation and differentiation. Similarly, Roy et al. (5) showed that reducing methionine availability during T cell stimulation leads to a reduced histone H3K4me3 and suppression of proliferation as well as a reduction in the expression of interleukin-17 (IL-17) and Interferon-gamma (IFN-γ) in mouse Th17 cells. Moreover, the authors showed that dietary restriction of methionine suppresses the expansion of pathogenic Th17 cells and slows disease progression in a mouse autoimmune disease model.

### SAM plays reciprocal roles in regulating T cell activation and effector function

Hot et al. (29) revealed that ethanol suppresses *MAT2A* expression in Jurkat and MOLT-4 CD4+ T cells, which leads to decreased SAM levels and increased T cell activation-induced apoptosis. This apoptotic phenotype can be prevented by pretreatment of T cells with SAM, indicating that SAM is required for T cell survival during activation. In contrast to this, SAM may have an opposite role in T cell function by suppressing CD8+ T cell-mediated anti-tumor immunity. T cells deficient in autophagy displayed reduced SAM level, which was associated with decreased global expression of H3K27me3, increased H3K4me3 density in the promotor regions of effector genes, and enhanced anti-tumor function of mouse CD8+ T cells (30). This implies that under certain metabolic situations, SAM may have suppressive functions *via* the regulation of histone methylation.

### SAH suppresses the activation of inflammatory CD4+ T cells while facilitating the differentiation of regulatory CD4+ T cells

SAH is generated from SAM by methyltransferases and is also a potent inhibitor of methyltransferases. SAH is rapidly converted to homocysteine by SAHH. However, in the presence of SAHH inhibitors, SAH accumulates, thus suppressing methylation reactions. Several studies have shown that SAHH inhibitors suppress CD4+ T cell activation and inflammation in tissue transplantation and arthritis mouse models (31–33, 48). In a more recent study, Huang et al. (34) found that SAHH inhibitors have reciprocal effects on inflammatory T cells and regulator T cells. The inhibitors reduced the frequencies of pro-inflammatory Th1 and Th17 cells. In contrast, inhibition of SAHH facilitated the differentiation of regulatory T cells in a cardiac transplantation mouse model. However, the effect of SAH accumulation on the epigenome and metabolism of T cells was not reported in these studies. Thus, further work is required to elucidate the mechanism by which SAH regulates the effector function of CD4+ T cells.

### Homocysteine promotes the proliferation and effector function of T cells through mitochondria-endoplasmic reticulum (ER) coupling

The regeneration of methionine from homocysteine by MTR or BHMT1 is the last step of the methionine cycle (**Figure 1**). Alternatively, homocysteine can exit the methionine cycle and be used for GSH synthesis, which is important for maintaining redox balance. Homocysteine promotes the proliferation and IFN-γ expression of mouse splenic T cells (35, 36). Feng et al. (35) suggested that this is mediated by enhanced mitochondria function. The authors found that homocysteine treatment during the activation of mouse splenic T cells increased mitochondrial activity and induced ER stress. Furthermore, homocysteine treatment increased the interaction between mitochondrial and ER networks in T cells (35). This interaction promotes mitochondrial function at the early stage of ER stress (49). On the other hand, treatment of a multiple sclerosis mouse model with calcitriol induced the expression of *BHMT1* in CD4+ T cells, reduced serum homocysteine level, increased the number of regulatory T cells, and prevented disease progression (37). Moreover, *in vitro* calcitriol treatment increased global DNA methylation in splenic CD4+ T cells, suggesting that reduction in homocysteine may be critical in stabilizing the phenotype of regulatory T cells (37).

## Tumor methionine metabolism drives T cell exhaustion

**Figure 2:**
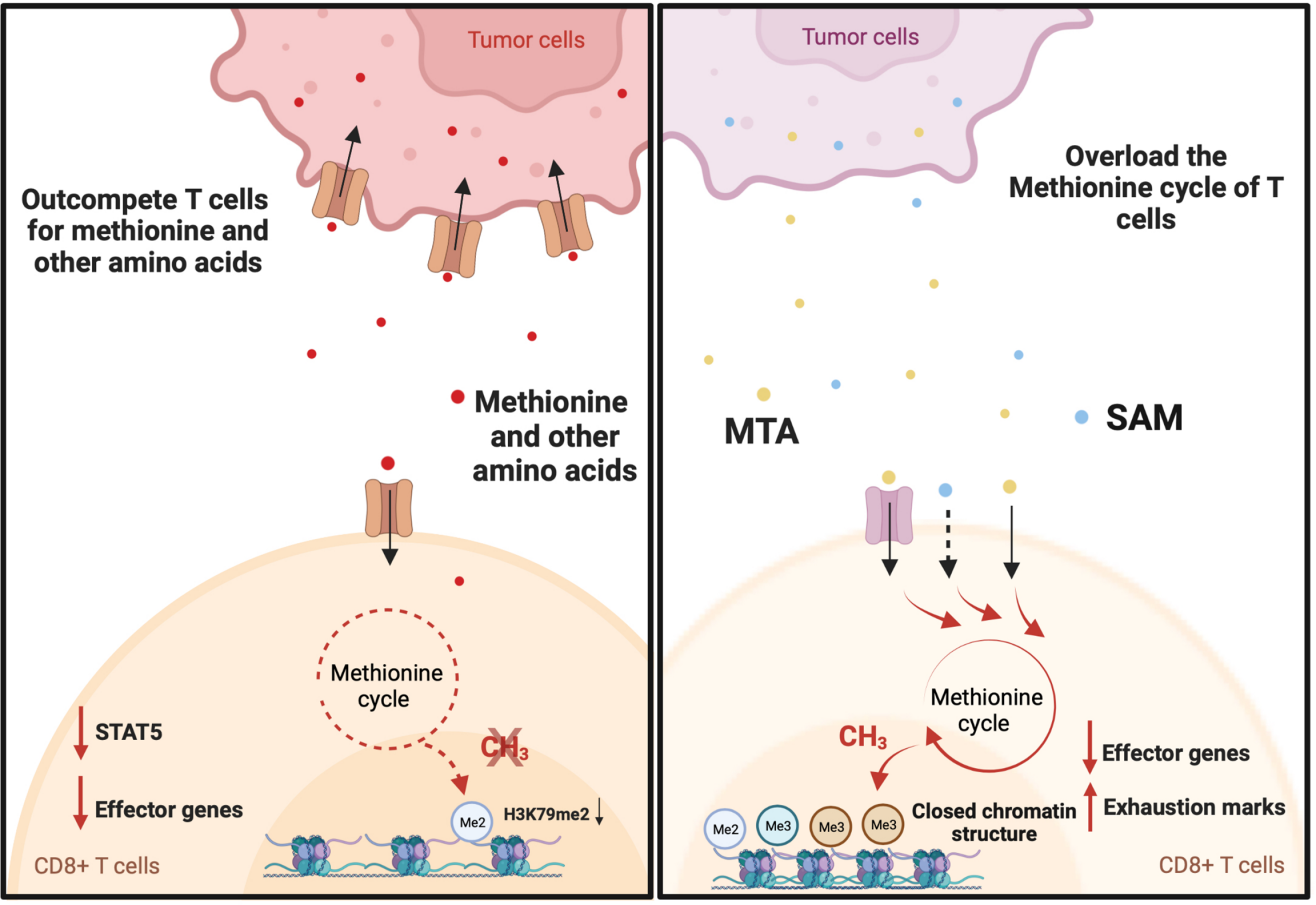
Tumor drives T cell exhaustion by manipulating the methionine cycle of T cells. Tumor cells promote T cell dysfunction by outcompeting T cells for methionine and potentially other amino acids. For example, B16F10 Tumor cells induce T cell exhaustion by outcompeting T cells for methionine uptake. This results in decreases in global H3K79me2 expression and the expression of Signal Transducer and Activator of Transcription 5 (STAT5). As a consequence, tumor-infiltrating CD8+ T cells displayed a reduction in the expression of interferon gamma (IFN-γ) and granzyme-B. On the other hand, hepatocellular carcinoma (HCC) tumors may produce immune-suppressive metabolites through methionine metabolism that help to induce T cell exhaustion. Elevated levels of S-adenosylmethionine (SAM) and 5′-methylthioadenosine (MTA) in HCC tumors are closely associated with T cell exhaustion. This may be explained by the immunosuppressive function of SAM and MTA. When supplemented *in vitro*, they promote the expression of exhaustion marks, inhibit IFN-γ expression, and cause a closed chromatin structure in human CD8+ T cells.

T cell exhaustion is a common state induced by chronic antigen exposure and in tumor-infiltrating lymphocytes. This exhausted state renders T cells dysfunctional and reversing or preventing this phenotype is a major goal in cancer immunotherapy (50). Exhausted T cells have a distinct epigenetic imprint compared with functional effector and memory T cells (51). Exhausted CD8+ T cells exhibit loss of chromatin-accessible regions (ChARs) at effector gene loci (*IFNG* and *GZMB*) and a gain of ChARs at exhaustion gene loci (*PDCD1*, HACVR2, and *BATF*) compared with non-exhausted T cells (51). In addition, T cell exhaustion in the context of chronic infection and cancer immunotherapy is associated with DNA demethylation at the *thymocyte selection-associated HMG BOX* (*TOX*) locus (52, 53), a gene that initiates and drives T cell exhaustion (54). Given that SAM and SAH play important roles in regulating the epigenetic landscape (9, 55), several reports have shown that tumor cells actively regulate the methionine metabolism of T cells to drive T cell exhaustion in the tumor microenvironment (**Figure 2**). There have also been studies demonstrating the key roles of other amino acids in the regulation of T cells (3, 5, 56–59). However, the role of these other amino acids on methylation has not yet been elucidated. Mouse melanoma cell line B16F10 overexpresses the methionine transporter SLC43A2 and consequentially tumor cells outcompete T cells for methionine uptake (38). This resulted in decreases in global H3K79me2 expression and the expression of Signal Transducer and Activator of Transcription 5 (STAT5). As a consequence, tumor-infiltrating CD8+ T cells displayed a reduction in the expression of IFN-γ and granzyme-B (38). Interestingly, methionine supplementation in patients with colorectal cancer increased the expression of H3K79me2 and phosphorylated STAT5 and enhanced the polyfunctional cytokine expression of CD8+ T cells (38). Together these data suggest that the exhaustion of CD8+ T cells is reversed by targeting the methionine metabolism of T cells. On the other hand, a study on 675 hepatocellular carcinoma (HCC) patients found that HCC tumors may produce immune-suppressive metabolites through methionine metabolism that help to induce T cell exhaustion (39). The study found that elevated levels of SAM and 5′-Methylthioadenosine (MTA), a downstream metabolite of SAM, in HCC tumors are closely associated with T cell exhaustion (39). Mechanistically, MTA suppresses the proliferation and effector function of CD8+ T cells through the inhibition of Akt signaling (60). In addition, *in vitro* treatment with SAM or MTA promoted the expression of exhaustion marks, inhibited IFN-γ expression, and caused a closed chromatin structure in human CD8+ T cells (39). Moreover, inhibiting tumor methionine metabolism by *MAT2A* knockout protected tumor-infiltrating CD8+ T cells from exhaustion in a syngeneic mouse tumor model (39). However, the mechanism by which SAM and MTA induce the exhaustion of CD8+ T cells is not fully understood.

## Perspective on methionine metabolism in T cell immunotherapy

Improving cancer immunotherapy by targeting the methionine cycle of T cells is an area that remains unexplored. Methionine restriction (MR) is a potent way to inhibit the methionine cycle. Many studies have demonstrated the suppressive effect of MR on tumor growth (61, 62). However, the effect of MR on T cells in the TME has not been fully explored. Given that a higher relative abundance of SAM is associated with reduced anti-tumor effector function of CD8+ T cells and reshaped histone methylation landscape that disfavors the expression of effector genes (30), the inhibition of the methionine cycle through MR may enhance the anti-tumor response mediated by CD8+ T cells through epigenetic reprogramming. On top of this, SAHH promotes the effector function of pro-inflammatory CD4+ T cells (34). Moreover, its product homocysteine promotes the proliferation and effector function of T cells (35, 36). Therefore, It is possible that SAHH overexpression can boost the anti-tumor effector function of T cells. Finally, targeting other enzymes in the methionine cycle may help to improve the anti-tumor function of T cells. For example, reducing the activity of MTs could block the transfer of methyl groups from SAM to DNA and histones (**Figure 1**). This may lead to an overall improvement in anti-tumor responses. Indeed, knockout of *DOT1L* reduced the global expression of H3K79me2 and increased the IFN-γ expression in mouse CD4+ T cells (63). In addition, *DNMT3a* knockout enhanced the expression of IFN-γ while reducing the expression of exhaustion mark Tim3 in CD8+ T cells (64). Moreover, a DNA demethylation reagent improved the persistence and anti-tumor function of CD8+ T cells in a PD1 blockade mouse model (64). Taken together, these findings suggest that targeting DNMTs and HMTs may be a useful strategy to improve the anti-tumor function of T cells.

While some of the aforementioned strategies to control methionine hold promise, the precise approach will depend on the baseline metabolic activity of the methionine and related cycles. Therefore, it is key that metabolite levels in these pathways are carefully evaluated in cancer patients and validated through functional assessments. Dietary modifications to alter methionine levels are an attractive strategy given the relative ease of delivery and the potential dual benefits it may provide (62).

**Table 1:** Summary of studies on the roles of methionine cycle metabolites in regulating T cell immunity.

Enzyme/metabolite	T cell subtype	Model/cancer type	Finding	Authors and year
Methionine	Mouse Th1 cells	*In vitro* generated and maintained mouse Th1 cells	T cell activation induces methionine uptake through the expression of SLC7A5 and SLC1A5, this is required for RNA methylation and the expression of H3K4me3 and H3K27me3 during T cell activation.	4
Methionine	Mouse Th1, Th17, andCD8+ T cells	*In vitro* generated and maintained mouse T cells and Experimental Autoimmune Encephalomyelitis (EAE) in C57BL/6 mice	Methionine restriction reduces the SAM level and the global expression of H3K4me3 in mouse T cells and inhibits the proliferation and Il-17 expression of Th17 cells and reduces disease onset and severity in an EAE mouse model.	5
MAT2A and SAM	Jurkat T cells and MOLT-4 CD4+ T cells	Immortalized human T cell lines	MAT2A inhibition increased the activation-induced cell death of CD4+ T cells. SAM supplement reduced ethanol-potentiated activation-induced cells death of T cells.	29
SAM	Mouse CD8+ T cells	*Atg5*+/- and *Atg5*-/- C57BL/6 mice implanted with e0771 (breast carcinoma) or Tramp-C2 (adenocarcinoma) tumors	SAM is negatively associated with the enrichment of H3K4m3 on the promoter regions of effector genes and with the anti-tumor function of CD8+ T cells.	30
SAHH	Mouse Th1 and Th2 cells	C57BL/6 mice challenged with ovalbumin	Inhibition of SAHH surpassed the proliferation, cytokine expression, and anti-OVA IgG production of Th1and Th2 cells.	31
SAHH	Mouse CD8+ T cells	*In vitro* maintained mouse mixed lymphocytes and tissue transplantation model (C57BL/6 to BALB/C)	Inhibition of SAHH suppresses the proliferation of CD8+ T cells *in vitro* and improves the survival of mice that received allografts.	32
SAHH	Mouse T cells enriched from lymph node cells	*In vitro* maintained T cells and chick type II collagen-induced arthritis in DBN/1 LacJ mice	Inhibition of SAHH suppresses the proliferation of T cells *in vitro* and prevents the onset and progression of arthritis in a mouse model.	33
SAHH	Mouse Th1, Th17, and Treg cells	Cardiac transplantation mouse model (BALB/C to C57BL/6)	Inhibition of SHHH reduces the frequency of Th1 and Th17 cells in CD4+ T cells and facilitates the differentiation of Treg cells in a cardiac transplantation mouse model.	34
Homocysteine	Mouse splenic T cells	Homocysteine treated C57BL/6 mice	Homocysteine treatment *in vivo* enhances the mitochondrial function of splenic T cells by increasing mitochondria-ER coupling.	35
Homocysteine	Mouse splenic T cells	*In vitro* maintained splenic T cells and hyperhomocysteinemia induced in Apo-E knockout mice	Homocysteine treatment enhanced Con A-induced proliferation of T cells *in vitro*. Induction of hyperhomocysteinemia in Apo-E knockout mice enhances the Con A-induced proliferation of T cells *in vitro*.	36
BHMT1	Mouse CD4+ T cells and Treg cells	Experimental Autoimmune Encephalomyelitis (EAE) in wild-type and *Vdr*-/- C57BL/6 mice	Calcitriol induces the expression of BHMT1 in CD4+ T cells, increases the number of Treg cells, and prevents disease progression in an EAE mouse model.	37
Methionine	Mouse and human CD8+ T cells	In vitro maintained mouse CD8+ T cells, C57BL/6 or BALB/C mice implanted with B16F10 (melanoma) tumors, and patients with colorectal cancer	Methionine supplementation delayed tumor growth in mice and increased the expression of H3K79me2, STAT5, and cytokines in CD8+T cells in tumorbearing mice and patients.	38
SAM and MTA	Mouse and human CD8+ T cells	Patient with hepatocellular carcinoma (HCC), *In vitro* maintained mouse CD8+ T cells, and C57BL/6 mice implanted with wild-type or MAT2A knockout Hep-55.1(HCC) tumors	Increased SAM and MTA levels in HCC tumors are positively associated with T cell exhaustion. SAM and MTA treatment *in vitro* induced exhaustion and caused a close chromatin structure in CD8+ T cells. Knockout of MAT2A in tumor cells protected CD8+ T cells from exhaustion in a tumor mouse model.	39

## Author contributions

All authors listed have made a substantial, direct, and intellectual contribution to the work, and approved it for publication.

## Funding

This study is funded by the University of Victoria Graduate Awards (TZ), the Canadian Institutes of Health Research (MOP-142351 and PJT-162279), IRiCOR, Ovarian Cancer Canada, and the US Department of Defense OCRP (W81XWH-18-1-0264).

## Conflict of interest

The authors declare that the research was conducted in the absence of any commercial or financial relationships that could be construed as a potential conflict of interest.

## Publisher’s note

All claims expressed in this article are solely those of the authors and do not necessarily represent those of their affiliated organizations, or those of the publisher, the editors and the reviewers. Any product that may be evaluated in this article, or claim that may be made by its manufacturer, is not guaranteed or endorsed by the publisher.
